# A true epidermotropic apocrine neoplasm in the form of perianal Paget’s disease: a case report

**DOI:** 10.1186/1752-1947-7-162

**Published:** 2013-06-20

**Authors:** Nikola Jankulovski, Liljana Spasevska, Vesna Janevska, Blagica Dukova

**Affiliations:** 1 University Clinic of Digestive Surgery, University “St Cyril and Methodius”, Skopje, Republic of Macedonia; 2 Institute of Pathology, Medical Faculty, University “St Cyril and Methodius”, Skopje, Republic of Macedonia

## Abstract

**Introduction:**

Extramammary Paget’s disease is an uncommon intraepithelial neoplasm that arises in areas rich in apocrine glands. Treatment includes wide surgical excision and nonsurgical modalities. We present the case of a patient with perianal Paget’s disease with no recurrent disease after wide surgical resection.

**Case presentation:**

Our patient was a 46-year-old man of Macedonian ethnicity who presented with a pruritic perianal lesion measuring up to 6cm without pain or bleeding. Two biopsies and a perianal wide surgical excision were performed. The tissue specimens were formalin-fixed and the paraffin-embedded samples analyzed according to standard histochemical and immunohistochemical procedures.

Surgical perianal skin excision revealed diffuse eczematoid, whitish plaques. Pathohistology showed Paget cells infiltrating his epidermis and adnexal epithelium, with ulceration. Immunohistochemical analysis revealed positive Paget cell expression for cytokeratin 7, epithelial membrane antigen, carcinoembryonic antigen, androgen receptor and human epidermal growth factor receptor 2, and negative expression for cytokeratin 20 and melan-A.

**Conclusion:**

Paget’s disease is a rare disorder that should be considered in the differential diagnosis of perianal lesions. Reporting cases of extramammary Paget’s disease is crucial for diagnostic guidelines and different therapeutic options.

## Introduction

Extramammary Paget’s disease (EMPD) is a rare cutaneous neoplasm that mainly affects the elderly. It predominantly involves apocrine gland-bearing areas, especially the vulva, scrotum and perianal region, but also the axilla, groin, thigh, external ear, umbilicus and nose [1]. Perianal Paget’s disease (PPD) was first described by Darier and Couillaud in 1893 [2]. There have been less than 200 reported cases in the literature, and the true incidence is difficult to estimate. PPD occurs either alone or in association with other adnexal malignancies or adenocarcinoma of the gastrointestinal tract. Clinical presentation includes nonspecific symptoms such as long-standing perianal eczema or pruritis, rash, bleeding, discharge and occasionally pain. Most of these lesions are first treated as benign dermatologic conditions with topical corticosteroids and antifungal therapy. The diagnosis is made by pathohistology and the treatment includes wide surgical excision and other treatment modalities. The rarity of the disease means that no large studies can be made, so it is crucial to report as many cases as possible in the literature to inform diagnostic guidelines and different therapeutic options. We present a case of PPD in a male patient with no other adnexal or gastrointestinal malignancy.

## Case presentation

A 46-year-old man of Macedonian ethnicity presented with a pruritic perianal lesion measuring up to 2cm without pain or bleeding. Our patient was diabetic and had a family history of diabetes and hypertension. Five months after his initial presentation, a colonoscopy and a biopsy were performed at Clinical Hospital Sistina - Adzibadem. The next month, our patient was admitted to our University Clinic of Digestive Surgery, where a physical examination revealed a perianal eczematous lesion measuring 6 × 4cm and enlarged inguinal lymph nodes. A second biopsy with a left lymphadenectomy was performed.

The tissue specimens were formalin-fixed and paraffin-embedded at our Institute of Pathology. We used a routine hematoxylin-eosin stain and performed additional histochemical and immunohistochemical analysis, including staining with Alcian blue and for cytokeratin (CK)7, CK20, epithelial membrane antigen, carcinoembryonic antigen, melan-A, androgen receptor and human epidermal growth factor receptor 2 (Her2/neu).

Microscopic analysis of the biopsy specimens showed large Paget cells with abundant pale cytoplasm, and large nuclei infiltrating the basal part and the whole thickness of the squamous epithelium and adnexal epithelium. Occasional cells had a signet-ring appearance. His inguinal lymph node revealed reactive lymphadenopathy. A diagnosis of EMPD was made.

**Figure 1:**
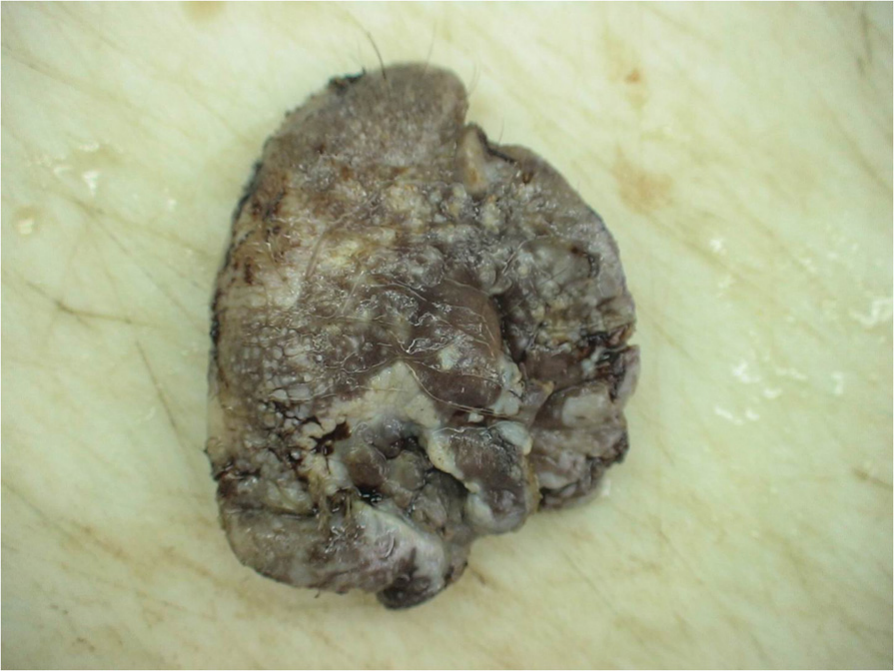
Gross view of formalin-fixed specimen.

**Figure 2:**
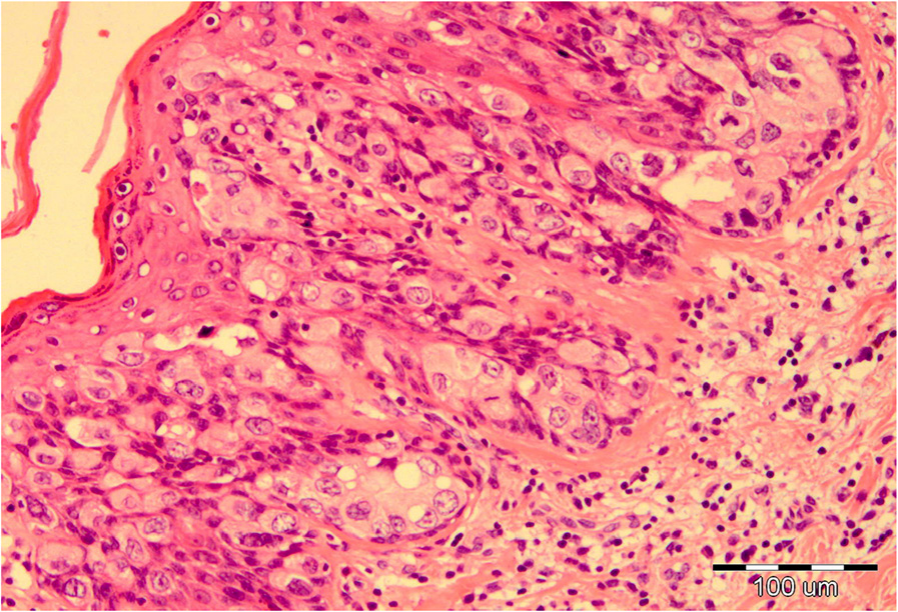
Paget cells with abundant amphophilic, clear cytoplasm and atypical oval nuclei, note the glandular cluster with central lumen in the lower epidermis, hematoxylin and eosin × 100.

**Figure 3:**
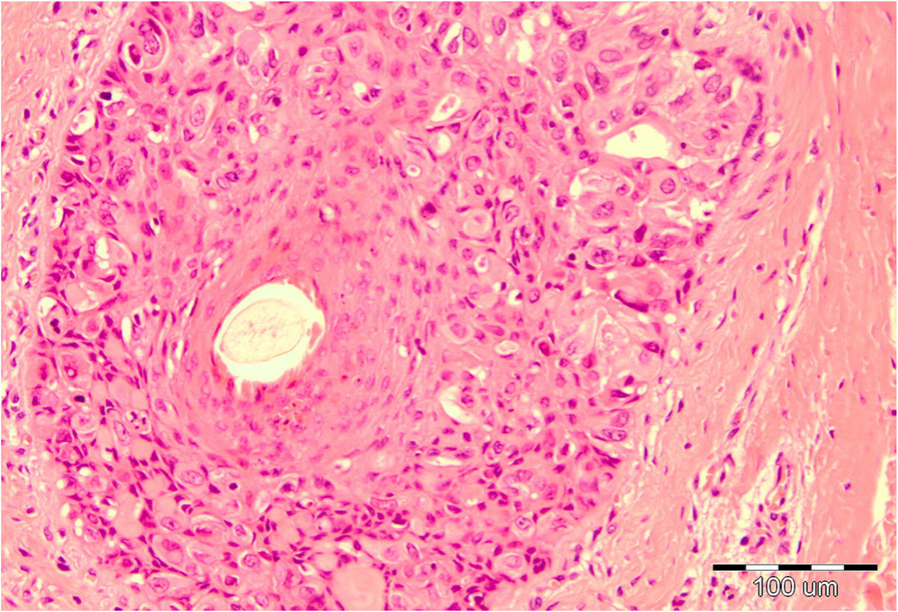
Paget cells located in hair follicle epithelium, hematoxylin and eosin × 100.

**Figure 4:**
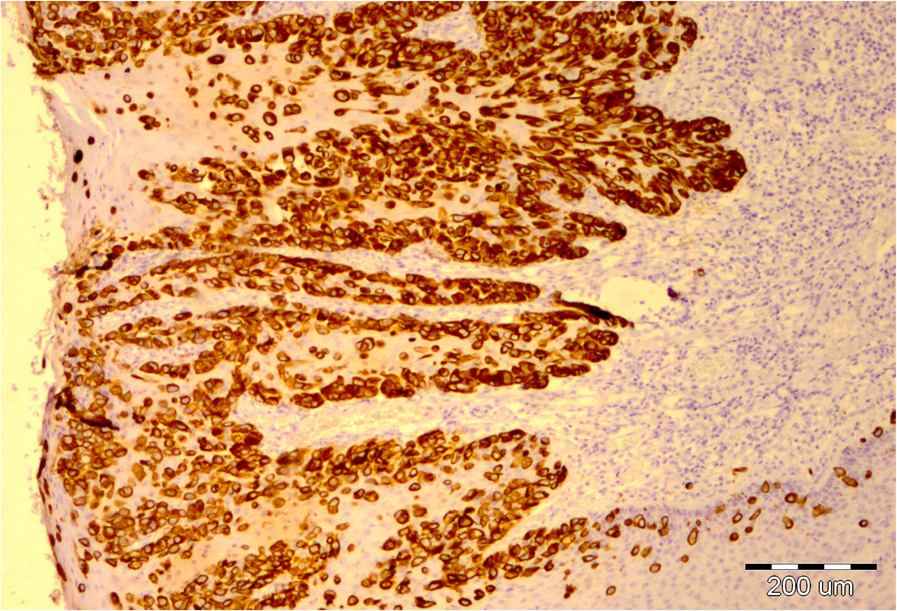
Intraepithelial Paget cells showing strong membrane staining for cytokeratin 7, × 40.

**Figure 5:**
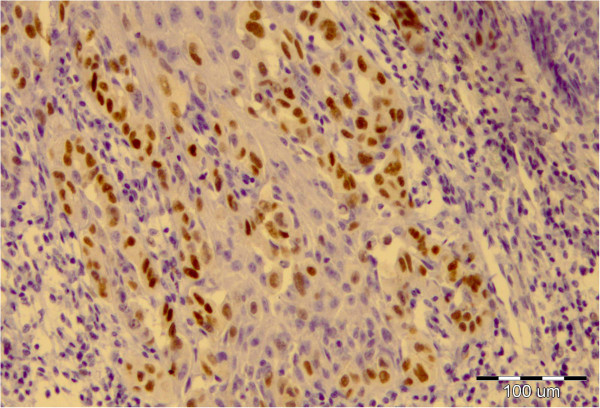
Nuclear positivity of Paget cells for androgen receptor, × 100.

**Figure 6:**
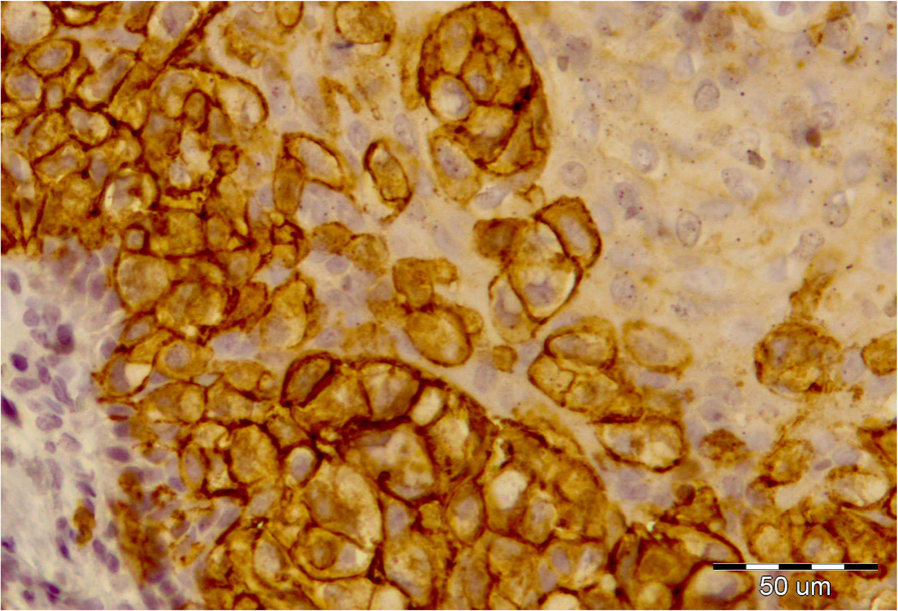
Complete membrane staining (3+) of Paget cells for human epidermal growth factor receptor 2, × 200.

**Figure 7:**
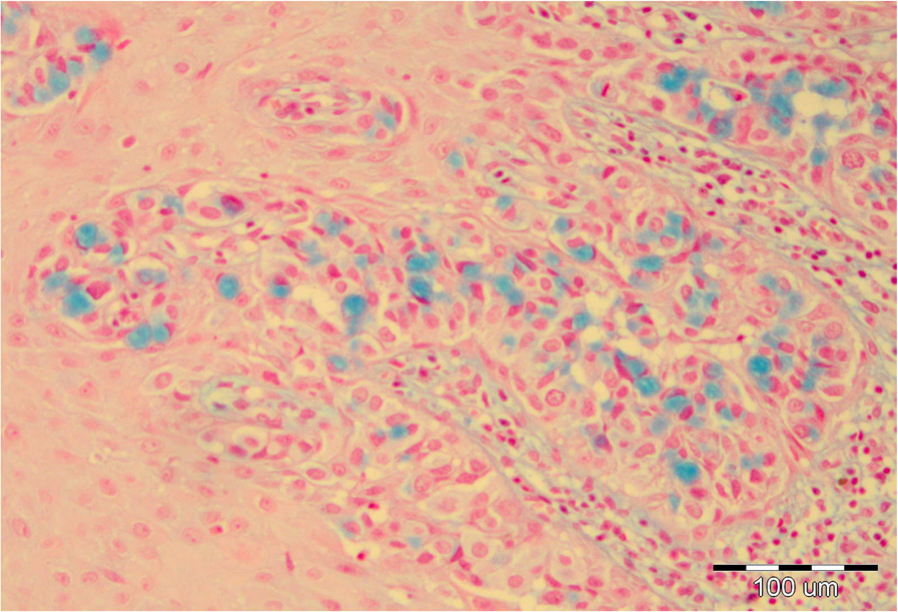
Intracytoplasmic mucine in Paget cells, Alcian blue × 100.

The perianal surgical skin excision measured 5.5 × 6.5 × 0.7cm and showed diffuse ulcerated eczematous plaques (Figure 1). Histopathology revealed identical Paget cells as viewed in the biopsy specimen, infiltrating the epidermis and adnexal epithelium with ulceration. In the basal epidermal layers there were some duct-like structures with small central lumina (Figures 2 and 3). There was intense mononuclear infiltrate in the dermal connective tissue. An immunohistochemical analysis on both biopsy and excision specimens revealed positive Paget cell expression for CK7, epithelial membrane antigen, carcinoembryonic antigen, androgen receptor and Her2/neu (Figures 4, 5 and 6), and negative expression for CK20 and melan-A. Intracytoplasmic sialomucin stained positive for Alcian blue (Figure 7). A diagnosis of primary *in situ* PPD was made.

**Figure 8:**
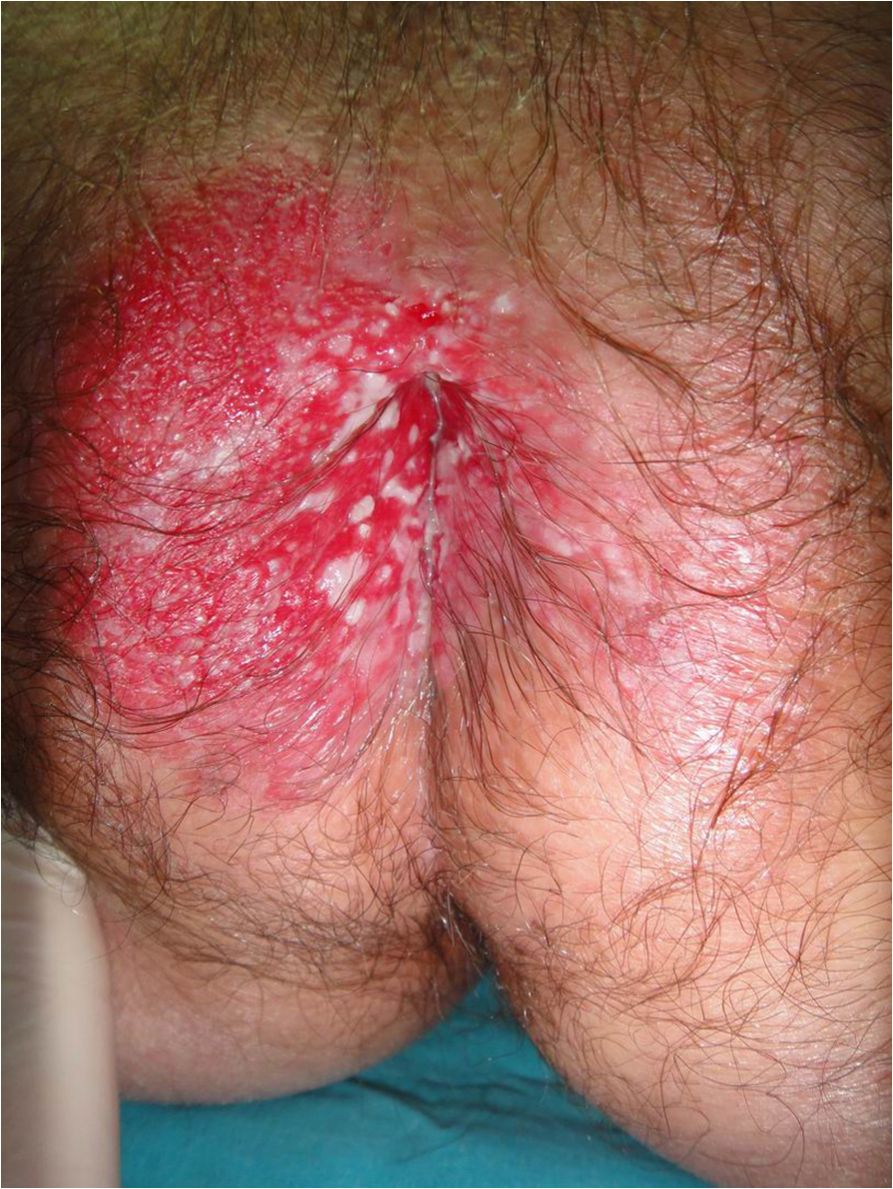
Clinical features of a patient with perianal Paget’s disease.

**Figure 9:**
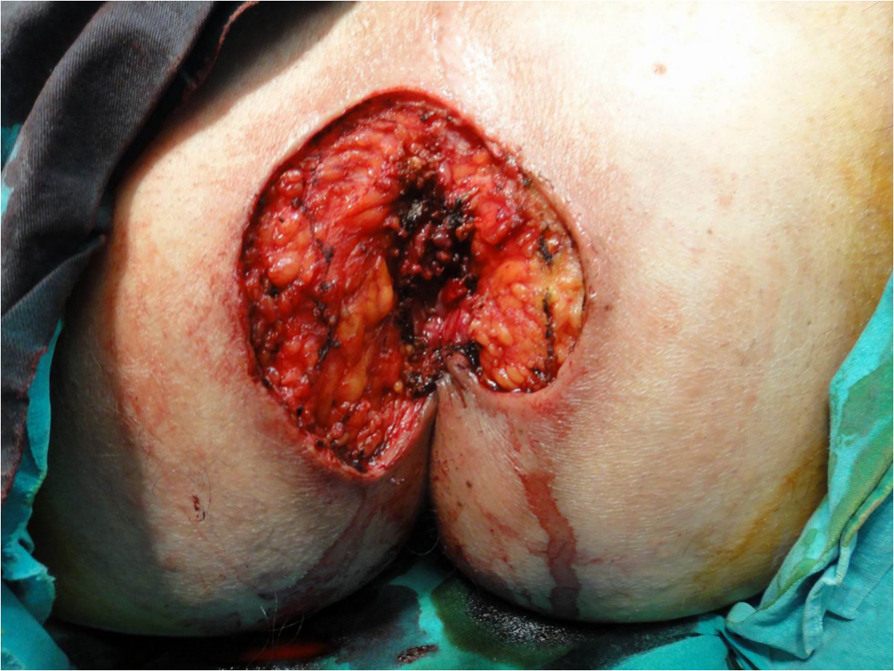
Post surgical excision.

After two biopsy findings of EMPD, a wide surgical excision was performed (Figures 8 and 9). The patient was discharged in good condition and advised to attend a follow-up examination. After six months, a check-up revealed his skin area to be disease free.

## Discussion

EMPD is a rare cutaneous intraepithelial adenocarcinoma arising in apocrine gland-bearing areas, particularly the perineum, vulva, axilla, scrotum, penis and groin. PPD mostly refers to intraepithelial carcinoma within 6cm of the anus and below the dentate line [3]. Patterns of involvement include an *in situ* epithelial form without associated carcinoma, an epithelial form with associated adnexal carcinoma, and association with visceral malignancy. Paget cells have the potential to invade the dermis and to metastasize. The anatomic site of the presenting EMPD is strongly related to the underlying visceral carcinoma in 86% of cases [4]. Our patient had an intraepithelial lesion without invasion of the dermis and no associated colorectal carcinoma.

Clinical presentation is often nonspecific and the diagnosis is frequently overlooked. Patients commonly have symptoms like pruritus, irritation and rash, although pain and bleeding may occur in long-standing cases. These lesions are typically erythematous, or whitish gray, dry and raised, but may turn into eczematoid, ulcerated, nodular or papillary forms [5-10]. It is unusual to make a diagnosis of EMPD clinically, so this condition is often first treated with topical corticosteroids and antifungal agents before a diagnosis is made by biopsy.

Differential diagnoses include Bowen’s disease, contact dermatitis, lichenoid lesions, psoriasis, melanoma, perianal Crohn’s involvement, mycosis fungoides, squamous cell carcinoma and tinea cruris.

Histopathology reveals large, round, clear-staining cells with abundant pale cytoplasm confined to the epidermis. The nuclei are large and situated in the periphery of cells. In the lower epidermal layers, glandular clusters can be seen that are absent in mammary Paget’s disease. Liu *et al.*[11] support the theory of two types of PPD with different immunoprofiles: primary cutaneous intraepithelial neoplasm (CK7-positive and CK20-negative), in which cells display sweat gland differentiation (gross cystic disease fluid protein 15-positive); and direct intraepithelial Pagetoid spread of anorectal adenocarcinoma (CK20-positive and CK7-negative, gross cystic disease fluid protein 15-negative). In one study, cells were androgen receptor-positive in 78% of patients and Her2/neu-positive in 52%. Coexpression, as in our patient, existed in 52% of patients [12].

Pathogenesis of PPD is controversial. Helwig and Graham [4] consider perianal and vulvar Paget’s disease to be a manifestation of a multicentric effect of an unknown carcinogenic stimulus on apocrine structures, epidermis and glandular elements of the rectum and urethra.

A patient diagnosed with EMPD needs an initial clinical assessment, evaluation of the extent of involvement of the lesion, and work-up for a possible underlying malignancy. A wide range of treatment modalities have been reported, including surgical and nonsurgical approaches. Surgical methods are still the mainstay in the management of PPD, including wide local excision with or without reconstruction and grafting, abdominoperineal resection, and Mohs micrographic surgery. Radiotherapy, chemo-radiotherapy and photodynamic therapy have been employed [6,7,13]. Despite varied modalities, local recurrence is a significant problem, to the extent of 33% [5]. The major factors in relapse and chronicity of the disease are multifocal involvement and difficulty in clinical delineation of cutaneous margins.

Prognosis depends on whether the disease extends beyond the epidermis and the adnexal epithelium. If it is associated with subjacent adnexal carcinoma or regional visceral carcinoma, the prognosis is poor. Survival of patients with *in situ* PPD is favorable.

Long-term follow-up of patients with EMPD is required to exclude the recurrence of the disease and development of an associated cancer. Follow-up should include a punch biopsy from the margin of the old perianal lesion once a year, in addition to a colonoscopy once every two years [1].

## Conclusion

We present the case of a patient with PPD treated with wide surgical excision. Our patient has demonstrated a disease-free period of six months. The diagnosis was made using pathohistology and immunohistochemistry. Although surgical excision is the proposed treatment of choice, other therapy modalities are used but not yet standardized. Long-term follow-up is essential to detect local recurrence and development of invasive Paget’s disease or anorectal carcinoma.

## Consent

Written informed consent was obtained from the patient for publication of this case report and accompanying images. A copy of the written consent is available for review by the Editor-in-Chief of this journal.

## Abbreviations

CK: cytokeratin; EMPD: extramammary Paget’s disease; PPD: perianal Paget’s disease.

## Competing interests

The authors declare that they have no competing interests.

## Authors’ contributions

NJ performed the biopsies and wide surgical excision, analyzed the data and followed-up the patient. LS, VJ and BD performed the histological examination of the specimens and interpreted the patient data. NJ and BD were major contributors in writing the manuscript. All authors have read and approved the final manuscript.
